# Endoplasmic Reticulum Stress and Ethanol Neurotoxicity

**DOI:** 10.3390/biom5042538

**Published:** 2015-10-14

**Authors:** Fanmuyi Yang, Jia Luo

**Affiliations:** Department of Pharmacology and Nutritional Sciences, College of Medicine, University of Kentucky, 132 Health Sciences Research Building, 1095 Veterans Drive, Lexington, KY 40536, USA; E-Mail: fyang3@uky.edu

**Keywords:** alcohol abuse, ER stress, development, fetal alcohol spectrum disorder, neurodegeneration, neuroprotection

## Abstract

Ethanol abuse affects virtually all organ systems and the central nervous system (CNS) is particularly vulnerable to excessive ethanol exposure. Ethanol exposure causes profound damages to both the adult and developing brain. Prenatal ethanol exposure induces fetal alcohol spectrum disorders (FASD) which is associated with mental retardation and other behavioral deficits. A number of potential mechanisms have been proposed for ethanol-induced brain damage; these include the promotion of neuroinflammation, interference with signaling by neurotrophic factors, induction of oxidative stress, modulation of retinoid acid signaling, and thiamine deficiency. The endoplasmic reticulum (ER) regulates posttranslational protein processing and transport. The accumulation of unfolded or misfolded proteins in the ER lumen triggers ER stress and induces unfolded protein response (UPR) which are mediated by three transmembrane ER signaling proteins: pancreatic endoplasmic reticulum kinase (PERK), inositol-requiring enzyme 1 (IRE1), and activating transcription factor 6 (ATF6). UPR is initiated to protect cells from overwhelming ER protein loading. However, sustained ER stress may result in cell death. ER stress has been implied in various CNS injuries, including brain ischemia, traumatic brain injury, and aging-associated neurodegeneration, such as Alzheimer’s disease (AD), Huntington’s disease (HD), Amyotrophic lateral sclerosis (ALS), and Parkinson’s disease (PD). However, effects of ethanol on ER stress in the CNS receive less attention. In this review, we discuss recent progress in the study of ER stress in ethanol-induced neurotoxicity. We also examine the potential mechanisms underlying ethanol-mediated ER stress and the interaction among ER stress, oxidative stress and autophagy in the context of ethanol neurotoxicity.

## 1. Introduction

Ethanol abuse is a leading preventable cause of death in the USA and is the fifth risk factor for premature death and disability, globally [1]. According to the report of National Institute on Alcohol Abuse and Alcoholism (NIAAA), 16.6 million adults (around 7% of the population in the USA) have an alcohol use disorder [2], which brings a huge economic and medical burden to the society ($ 223.5 billion in 2006) [3]. Chronic ethanol consumption and binge drinking have adverse effects on multi-organs, including the liver, pancreas, heart, and central nervous system (CNS). Ethanol abuse causes profound damage to both the mature and developing brain [4,5,6]. Offspring from mothers consuming ethanol during pregnancy may develop fetal alcohol spectrum disorders (FASD); fetal alcohol syndrome (FAS) is the severest form, which includes a group of pathological conditions, such as facial abnormalities, growth retardation, poor memory, vision and hearing problems, problems of heart, kidney and bone, and intellectual and behavioral deficits, ranging from mild to severe [7]. FASD is the top preventable cause of mental retardation [7]. However, the mechanisms underlying alcohol-induced brain damage are unclear.

Although pathological development caused by ethanol abuse is organ-, region-, and age-specific, there may be some common mechanisms accountable for ethanol-induced organ damages. It is believed that inflammatory reactions and cytokine production mediate the adverse effect of ethanol on multiple organs [8] and genes related to neuronal inflammatory responses might be the potential therapeutic targets for ethanol-induced neuronal toxicity [9]. Oxidative stress has been proposed as a major contributor to ethanol-induced multi-organ damage, including the CNS pathogenesis [10,11,12,13]. Oxidative stress occurs in response to the imbalance between production and removal of the reactive oxygen species (ROS) in cells [14]. It is known that ROS are continuously generated in each step of ethanol metabolism and can be removed efficiently by evolutionarily developed antioxidants [14]. However, the disruption of this homeostasis by severe environmental insults, such as excessive ethanol exposure, leads to pathological oxidative stress and subsequent cellular damage. Recently, microRNA, such as microRNA-29b, has also been shown to regulate ethanol-induced cerebellum neuronal death [15].

In addition, studies suggest that ER stress may play an important role in ethanol-induced organ damage. Abnormal ER protein loading and modification can be induced by a variety of stimuli, which disrupt the intracellular homeostasis. However, the role of ER stress in ethanol-induced neurotoxicity has received little attention. Further, ER as the key apparatus of protein folding and modification is closely related to mitochondria, the energy center of redox reactions, leading to the cross-talk between ER stress and oxidative stress. ER stress can also induce autophagy, a degradation machinery to maintain cell homeostasis. This review will discuss the involvement of ER stress in ethanol-induced brain damage and potential underlying mechanisms including the relationship between ER stress, oxidative stress and autophagy.

## 2. ER Stress and CNS Pathology

The physiological or pathological signals disturb protein post-translational modifications, such as folding, glycosylation, *etc.*, causing accumulation of unfolded or misfolded proteins in the ER lumen and inducing ER stress. ER stress subsequently triggers unfolded protein response (UPR). UPR, which is initiated as a conserved adaptation mechanism attenuates ER stress by decreasing ER loading (shutting down or inhibiting the protein synthesis) and increasing ER capacity (increased function and efficiency of chaperone proteins) to improve accurate protein folding. During ER stress, chaperone protein, glucose-regulated protein 78 (GRP78) is dissociated from the binding sites on the lumen tails of three major transmembrane proteins, pancreatic endoplasmic reticulum kinase (PERK), inositol-requiring enzyme 1 (IRE1), and activating transcription factor 6 (ATF6), leading to their oligomerization or translocation-mediated activation. Activation of IRE1 exposes its endoribonuclease domain which is responsible for processing the mRNA of X box protein-1 (XBP1), turning on XBP1 protein translation. XBP1 is a transcription factor and can induce transcription of proteins involved in ER retrograde transportation and protein degradation [16]. Activated PERK phosphorylates and inactivates eukaryotic initiation factor 2α (EIF2α), halting protein translation and reducing ER protein loading without affecting the production of ATF4 which is a transcriptional regulator of UPR genes [16]. ATF6 is cleaved and activated in the Golgi complex, and then translocates to the nucleus working together with IRE1 to upregulate XBP1 [17]. If the ER homeostasis cannot be restored by the adaptation, alarm stage is induced, which is characterized by the activation of stress signaling pathways, such as c-Jun N-terminal kinase (JNK), Mammalian p38 mitogen-activated protein kinases (p38 MAPK), and nuclear factor κB (NF-κB) pathways and the production of inflammatory cytokines [16]. Cell death will occur if pathological stimuli persist, characterized by caspase activation, especially caspase 12, and C/EBP homologous protein (CHOP) expression [16].

UPR genes may participate in the regulation of developmental events. For example, Zhang and colleagues discovered a higher baseline level of calreticulin, GRP78, GRP94, ER protein 57, and PDI in embryonic mouse brains [18]. They also found the dominant expression of spliced XBP1 mRNA and partially glycosylated ATF6 in embryonic brain [18]. Another report shows that UPR markers (PERK, GRP78, CHOP, IRE1, ATF4, ATF6, and XBP1) are inducible in oocytes and periimplantation embryos and are very important for placentation and early organogenesis [19].

Altered regulation of ER stress has been also revealed in various forms of CNS injury, including brain ischemia, traumatic brain injury, spinal cord injury, epilepticus, and neurodegeneration, including Parkinson’s disease (PD), Alzheimer’s disease (AD), Huntington’s disease (HD), and amyotrophic lateral sclerosis (ALS) [20]. Take PD and AD, the age-dependent neuronal degeneration, as the example. The expression of ER stress markers, such as GPR78, PERK, EIF2α, and IRE1α, are altered in AD patient samples [21,22,23]. β-amyloid extracellular accumulation and tau protein hyper-phosphorylation, the two key players in AD pathogenesis, are positively associated with the ER stress markers [20]. β-amyloid is produced under physiological condition, while its secretion and lysosomal localization is up-regulated under cellular stress [24]. *In vitro* experiments showed that β-amyloid increases the expression of GRP78 [25], CHOP, and active caspase 12 in neurons [26], while it decreases the stable ER association with microtubules leading to ER collapse [27]. Moreover, the tau protein is co-localized with active PERK, the upstream ER stress initiator, and the hyperphosphorylation of tau can stimulate activation of PERK and EIF2α, as well as the expression of XBP1 and CHOP [28]. PD is mainly caused by loss of dopaminergic neurons in *substantia nigra pars compacta.* The loss of dopaminergic neurons may be caused by intracellular accumulation of Lewy bodies, the α-synuclein fibrillary aggregates. The association between ER stress and PD has been revealed by evidence from clinical and animal model studies. For instance, up-regulation of ER stress markers, such as active PERK and EIF2α, are observed in PD patients [29]. In parallel, the expression of GRP78, XBP1, CHOP, and ATF4 is up-regulated in a transgenic mice model overexpressing α-synuclein [30]. Studies support that overexpression of α-synuclein can disrupt ER-Golgi trafficking, therefore, leading to subsequent ER stress [31,32]. Increased mutant Huntington protein (HTT) in Huntington’s disease and accumulation of superoxide dismutase (SOD1) in ALS have been shown positively correlated with ER stress [20]. It is also true that ER stress in turn promotes the accumulation of α-synuclein aggregates, suggesting the adverse effects of communication between ER stress and α-synuclein aggregation [33]. ER stress is also involved in other neuropathy. For example, ER stress-induced apoptosis may be responsible for post-traumatic stress disorder in rat hippocampus; the cell death is accompanied by increased expression of GRP78 and caspase 12, along with an increased intracellular calcium level [34]. Calreticulin, CHOP, XBP1s and p-EIF2α are significantly increased in rat and human inflammatory demyelination, suggesting the occurrence of ER stress [35]. Previous studies of *status epilepticus* in rat brains have shown that ER stress induces neuronal death in a temporal and spatial specific pattern [36].

Targeting ER stress may provide potential therapeutic approaches for neurodegeneration and brain damage. For example, ginsenoside Rb1, a natural ingredient in ginseng, can attenuate high glucose-induced injury in rat hippocampal neurons [37]. The data showed that Rb1 down-regulates the protein level and activation of PERK and the downstream effector—CHOP, suggesting a possible neuroprotective role of Rb1 by alleviating ER stress [37]. Additionally, activation of glycogen synthase kinase 3β (GSK-3β), another modulator of CHOP in neurons, is inhibited [37]. Similarly, in a traumatic brain injury (TBI) rat model, docosahexaenoic acid (DHA) treatment not only attenuates activation of EIF2α, ATF4, inwardly rectifying potassium channel (IRK1) and CHOP, it reduces abnormal ubiquitin aggregates, decreases amyloid precursor protein (APP) and phosphorylates tau in the frontal cortex; it also improves the recovery of sensorimotor neuronal function [38]. These evidences suggest a potential connection between inhibiting ER stress and beneficial effects in neuronal injury. On the contrary, some studies showed a protective role of ER stress. For example, salubrinal, an inhibitor of ER stress, suppresses autophagy activation and eliminates the neuroprotection induced by brain ischemic preconditioning in permanent focal ischemia [39]. Similarly, insufficient UPR leads to cerebellar granule cell degeneration in murine congenital disorders of glycosylation, indicating that ER stress response in the early adaptive stage is beneficial in restoring physiological environment and neuronal function [40]. Other convincing evidence is that the expression of human wild-type Leucine-rich repeat kinase 2 (LRRK2), the most frequent mutated gene in PD patients [41], plays a protective role on α-synuclein-induced neurotoxicity in *C. elegans*, functioning through up-regulated GRP78 [42]. The expression of G2019S mutant LRRK2 in *C. elegans* leads to aging-associated neurodegeneration and p38 MAPK activation-mediated cell death [42].

## 3. ER Stress and Ethanol-Induced Organ Damage

The involvement and critical role of ER stress in ethanol-induced organ damage has been demonstrated in the liver, pancreas, and heart. Ethanol exacerbates ER stress responses and promotes liver damage and dyslipidemia in fatty liver diseases [43]. Ethanol dose-dependently enhances palmitic acid (PA)-induced ER stress and apoptosis in primary rat hepatocytes and in a high-fat diet-treated mouse model [44]. Genetically manipulating genes involved in ER stress-induced apoptosis, such as CHOP knockout mice, attenuates liver damage and dyslipidemia in mice caused by ethanol exposure [44,45]. ER stress can induce a fibrigenic response in liver stellate cells through autophagy, which can be attenuated by blocking IRE1 [46]. Apart from the liver, other organs, such as heart and pancreas, are also affected by ethanol-induced ER stress [47,48]. Chronic ethanol exposure profoundly enhances cardiac hypertrophic remodeling, myocardial dysfunction, oxidative stress and ER stress, suggesting that ER stress might be involved in ethanol-induced heart damage [48]. Ethanol-induced ER stress is related to the dysfunction of pancreatic β-cells in mice [49]. Intriguingly, XBP1s, a downstream factor in UPR, protects the pancreas against ethanol-induced damage [50]. XBP1s-deficient mice display attenuated UPR and exacerbated apoptosis in pancreas in response to ethanol exposure [50].

## 4. ER Stress and Ethanol-Induced Brain Damage

The CNS is particularly vulnerable to ethanol-induced damage. Pathological alterations in brain structure and neuronal function have been observed in patients and animal models [51,52,53,54]. Ethanol causes significant damage in several subdivided areas of the brain, such as cerebellum, hippocampus, and frontal cortex. Binge drinking causes long-lasting effects on emotional and memory deficits, suggesting the functional and structural alteration in the hippocampus in adolescent female mice [54].

We have been studying the role of ER stress in ethanol-induced CNS damage using both *in vitro* and *in vivo* models. Using SH-SY5Y neuroblastoma cells and primary cerebellar granule neurons as *in vitro* models, we demonstrate that exposure to ethanol alone has little effect on the expression of markers of ER stress; however, ethanol dramatically enhances the expression of GRP78, CHOP, ATF4, ATF6, and phosphorylated PERK and EIF2a when applied with tunicamycin and thapsigargin [55]. Ethanol exacerbates tunicamycin- or thapsigargin-induced ER stress as early as 6 h after treatment and the effect persists for at least six more hours [55]. Ethanol rapidly causes oxidative stress in cultured neuronal cells; antioxidants, such as glutathione (GSH) and *N*-acetyl-l-cysteine (NAC), blocks ethanol’s potentiation of ER stress and cell death, suggesting that the ethanol-promoted ER stress response is mediated by oxidative stress [55]. CHOP is a pro-apoptotic transcription factor. CHOP deficient cells show attenuated cell death in response to ethanol, suggesting that CHOP may play an important role in ethanol-promoted cell death [55]. Thus, ethanol-induced death of neuronal cells may be mediated by the interaction of oxidative stress and ER stress.

**Figure 1:**
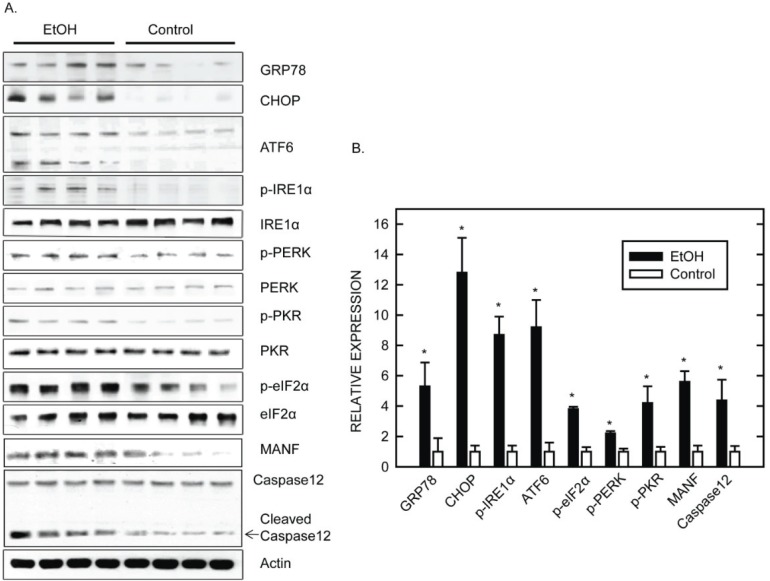
Ethanol induces ER stress in the developing brain, adapted from data published by Ke, Z. *et al.* [5]. (**A**) mice of postnatal day seven (PD7) were subcutaneously injected with ethanol (2.5 g/kg) or saline at time 0 and 2 h. Eight hours after the first injection, the cerebral cortex was harvested and the expression of ER stress markers was analyzed with immunoblotting. N = 4 for each group; (**B**) The protein expression was quantified by densitometry, normalized to the level of actin and presented as mean ± SEM [5].

**Figure 2:**
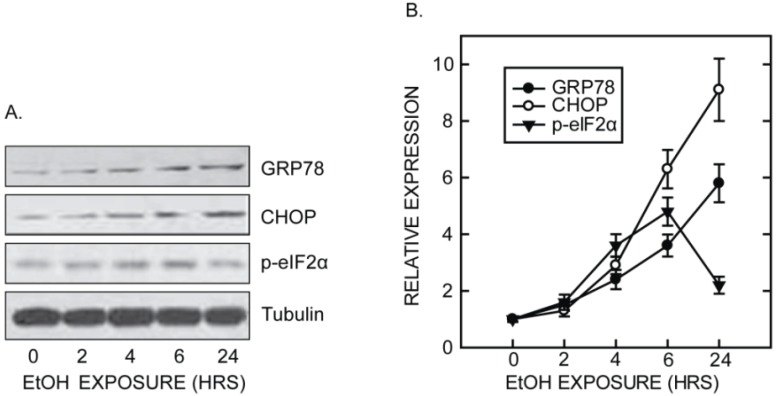
Time course of ethanol-induced ER stress in the developing brain adapted from data published by Ke, Z. *et al.* [5]. (**A**) Mice PD7 were subcutaneously injected with ethanol or saline as described in Figure 1. At 2, 4, 6, and 24 h after ethanol injection, animals were sacrificed and protein from the cerebral cortex was extracted. The expression of GRP78, CHOP, p-eIF2α, and tubulin was analyzed with immunoblotting; (**B**) the protein expression was quantified by densitometry, normalized to the level of actin and presented as mean ± SEM [5].

We have used a well-established third trimester equivalent mouse model of ethanol exposure [56] to further investigate the effect of ethanol on the developing brain. In this model, ethanol causes a wide spread neuroapoptosis in the brain of postnatal day seven (PD7) mice [5]. We show that ethanol induced UPR in the brain which is indicated by the significant upregulation of ATF6, CHOP, GRP78, IRE1α, PERK, p-EIF2α, cleaved caspase-12, and mesencephalic astrocyte-derived neurotrophic factor (MANF) (Figure 1) [5]. The up-regulation of MANF has been observed in neuronal injury and degeneration, such as cerebral ischemia [57,58], and so far is recognized as a machinery-protecting neuron from severe impairment by alleviating ER stress [59]. Decreased expression of MANF is related to neuronal degeneration [60]. In our experiments, the increase of UPR markers is evident at 4 h following ethanol exposure and is sustained for over twenty-four hours (Figure 2) [5]. However, unlike *in vitro* studies, knocking out CHOP does not offer protection against ethanol-induced neurodegeneration in the developing brain [5]. That is, ethanol-induced neurodegeneration is similar between wild-type and CHOP-deficient mice. As a result, the role of CHOP in ethanol-induced neurodegeneration in the context of ER stress warrants further research. Immunostaining revealed that ER stress markers, p-EIF2α, CHOP, and cleaved caspase-12 are colocalized with neurons rather than astroglias, indicating it is neurons undergoing UPR after ethanol treatment [5]. In addition to neurons, Goral and Meyer showed that ethanol can induce CHOP and XBP1 expression in BV2 microglia which attenuated microglia inflammation response (NO production) induced by LPS [61]. Therefore ethanol-induced ER stress could possibly occur in both neurons and glial cells.

The sensitivity to ethanol-induced brain damage depends on the status of neurodevelopment. We showed that ethanol induced a wide-spread neuroapoptosis in postnatal day four (PD4) C57BL/6 mice, but has little effect on the brain of PD12 mice [4]. We analyzed the expression profile of genes regulating apoptosis, and the pathways of UPR and autophagy during these ethanol-sensitive and resistant periods (PD4 *versus* PD12) using PCR microarray [4]. The expression of pro-apoptotic genes, such as cleaved caspase-3, is much higher on PD4 than PD12; in contrast, the expression of genes that regulate UPR and autophagy, such as atf6, atg4, atg9, atg10, beclin1, bnip3, cebpb, ctsb, ctsd, ctss, grp78, ire1α, lamp, lc3 perk, pik3c3, and sqstm1 is significantly higher on PD12 than PD4 [4]. These results suggest that the vulnerability of the immature brain to ethanol could result from high expression of pro-apoptotic proteins and a deficiency in the stress responsive system, such as UPR and autophagy. Indeed, our study supports that immature brain is much more vulnerable to ER stress-induced brain damage than a mature brain (Wang *et al.*, 2015) [62].

Until now, the information regarding ethanol’s effect on ER stress in the adult brain is very limited. Using a model of pre-exposing rats with chronic ethanol treatment followed by *ex vivo* ER stress induction by thapsigargin in brain slices, Dlugos showed that ethanol pre-exposure significantly increases the expression of caspase 12 and ATF6 in the brain slices [6]. This result is consistent with our previous *in vitro* study showing that ethanol exacerbates tunicamycin- or thapsigargin-induced ER stress in cultured neurons [55].

It is currently unclear the exact role of ER stress response or UPR in ethanol-induced brain damage. Based on the studies performed in other organ systems, initial UPR may be beneficial in alleviating ethanol neurotoxicity. In addition to UPR, ethanol may activate autophagy through ER stress [63,64]. Autophagy regulates the degradation of misfolded protein and damaged organelles through the lysosomal-system which is characterized by formation of autophagosomes. Autophagy is proposed as a protective pathway for cell survival under overwhelmed ER stress [65]. We have previously shown that the expression genes regulating both UPR and autophagy pathways is higher during the ethanol-resistant period (PD12) compared to the ethanol-sensitive period in mice (PD4) [4]. Consistently, application of rapamycin, an activator of autophagy, attenuates ethanol induced-neuronal death [64]. On the contrary, wortmannin, an inhibitor of autophagosome formation, increases neuroapoptosis induced by ethanol exposure [64]. These results suggest that autophagy is a protective response to mitigate ethanol-induced CNS damage. Both oxidative stress and ER stress can induce autophagy in different cell types [63,66]. In neurons, mitochondrial damage and NADPH oxidase activation by oxidative stress triggers autophagy, which can be subsequently inhibited by antioxidant application [63,64]. Disruption of intracellular calcium homeostasis and activation of certain kinases and transcription factors by ER stress leads to autophagy [63,66]. Taken together, both ER stress and oxidative stress may initially stimulate autophagy which attempts to eliminate damaged protein and organelles, offering protection against ethanol’s insult. However, when sustained and intensive insults exceed the capacity of these protective responses, neurons undergo degeneration. This might be due to the activation of NF-κB inflammatory pathway [67]. Evidence has shown that each UPR inducer (ATF6, IRE1, PERK) is able to activate NF-κB, leading to the excessive production of pro-inflammatory cytokines, such as TNFα, IL1β, and IL6, which could be responsible for neuronal degeneration [67].

## 5. Mechanisms Underlying Ethanol-Induced ER Stress

The mechanisms underlying ethanol-induced ER stress in the CNS remain unclear. Studies on other organs, such as liver, heart, and muscle, offer several possibilities.

Acetaldehyde, an intermediate of ethanol metabolism generated by alcohol dehydrogenase (ADH), can interact with nucleophilic groups in proteins under physiological conditions and generate unstable acetaldehyde adducts. These adducts can induce immune responses which subsequently leads to conformational change of the targets and pathological protein degradation [8]. Acetaldehyde adducts are found in various diseases, such as atherosclerosis and ethanol-induced cardiac remodeling and dysfunction, and are correlated with increased ER stress responses [68,69]. The casual relationship between acetaldehyde adducts and ER stress has been revealed by decreased expression of GRP78 upon ADH inhibition in perfused rat liver system [70]. Acetaldehyde adducts are present in the brain of an addicted alcohol user who died after drinking [71] and are also found in degenerated cortical neurons of chronic ethanol-fed mice [72], which is co-localized with the ER stress response region in the alcoholic model [5], suggesting a possible association between acetaldehyde adducts and ER stress.

ER stress can also be triggered by the disruption of intracellular calcium homeostasis which is critical for ER protein folding and modification [8]. Ethanol metabolites have been shown to deteriorate the function of both skeletal muscle fibers [73] and ventricular myocytes [74] in rats, which is probably due to the interference of ER calcium homeostasis [75]. It has been shown that ethanol disrupts intracellular calcium homeostasis in neurons too [76,77]. Ethanol dose-dependently induces cell death in primary culture of cerebellum granule neurons through increasing intracellular calcium release [77]. Interestingly, *in vitro* chronic ethanol treatment on septal neurons of rat fetuses, which were derived from the mothers pre-exposed to chronic ethanol feeding, can alter the calcium homeostasis and adversely affect calcium-involved cell functions, including neuronal migration, neurite outgrowth and synaptogenesis [76]. The ratio of phosphoatidylcholine (PC) and phosphatidylethanolamine (PE) which are the major compositions of the ER membrane is altered by ethanol, leading to compromised sarco/endoplasmic reticulum calcium ATPase (SERCA) activity and subsequent perturbed calcium homeostasis in the ER [78]. Further, ethanol-induced excessive ROS production from mitochondria can interfere in the function of ER chaperones and ER calcium channels, leading to the calcium efflux to the cytosol [79]. The vicious cycle goes on when up-regulated intracellular calcium level promotes mitochondria to produce more ROS which further perturbs ER calcium homeostasis [79]. It is therefore likely that ethanol may induce ER stress by perturbing intracellular calcium homeostasis in neurons.

Oxidative stress is involved in ethanol-induced organ damage [12]. Ethanol-induced oxidative stress has been observed in both clinical studies and animal models [14]. A clinical study showed that chronic alcoholic patients have significantly increased free radicals without signs of hepatic lesion [12]. In a primate model, prolonged ethanol feeding is shown to cause hepatopathy, including fatty liver disease, inflammatory responses, cell apoptosis and necrosis, liver fibrosis, and sclerosis [80]. Multiple studies have shown that the mitochondria, which contains a number of enzymes involved in redox-oxidative reaction, undergoes structural alteration upon ethanol exposure, indicated by enlargement, swelling, shortening, and decreased matrix granules and intra-mitochondria crystalline inclusions [81,82,83]. It is also interesting to note that in an ethanol feeding rat model, days before the mitochondria is affected, morphological alterations are observed in the ER, which are evident by increased vesicle structure along with smooth membrane proliferation, suggesting that ER malfunction occurs before oxidative stress [81].

Oxidative protein folding in the ER, such as formation of disulfide bonds, requires maintaining an exclusive redox microenvironment which accounts for 25% of ROS produced by the whole cell [84]. Ethanol induces overwhelming ROS production by the mitochondria, including H_2_O_2_, superoxide, and lipid peroxide, which impairs the redox condition in ER and leads to ER stress [12]. Consistently, ER stress is positively correlated with the oxidation level of glutathione, which maintains the ER redox environment, in pancreatic cells of ethanol-treated rats [47]. Antioxidant NAC is able to attenuate ethanol-induced ER stress and neuronal death, suggesting that ER stress in the brain may be initiated by oxidative stress [55]. On the other hand, ER stress may promote oxidative stress. For example, in rats chronically exposed to ethanol, ER stress occurs earlier than oxidative stress in liver cells [81]. Persistent ER overload may activate an inflammatory response and disrupt calcium homeostasis and redox microenvironment, leading to mitochondrial dysfunction and ROS production [16]. ER calcium depletion through Inositol trisphosphate (IP3) receptors, caused by ER stress, leads to mitochondria hyperactive calcium intake which increases ROS generation and apoptosis [75,85,86]. Ethanol is also shown to induce iron overload and impair mitochondrial iron-sulfur clusters, causing excessive oxidative stress which may promote ER stress responses [87,88,89,90]. Since ER stress can affect the expression of hepcidin [91], a key regulator of iron homeostasis [92], a positive feedback may further enhance the effect of ethanol, inducing ER overload and mitochondrial dysfunction. Similarly, interfering with the function of ER chaperones and ER calcium channels by ROS leads to the calcium efflux to the cytosol which in turn exacerbates the oxidative stress generated by mitochondria [79].

## 6. Conclusions

There is convincing evidence showing that ethanol may induce ER stress in neurons, particularly in the developing CNS. However, the exact role of ER stress or UPR in ethanol-induced brain damage remains unclear. Initial UPR may be beneficial in alleviating ethanol neurotoxicity. There are several potential mechanisms accountable for ethanol-induced ER stress in neurons, these include the production of acetaldehyde adducts, oxidative stress, and disruption of intracellular calcium homeostasis. There is considerable interaction among ER stress, oxidative stress, and autophagy. However, how this interaction contributes to ethanol neurotoxicity warrants further study.
